# Preliminary Study: Learning the Impact of Simulation Time on Reentry Location and Morphology Induced by Personalized Cardiac Modeling

**DOI:** 10.3389/fphys.2021.733500

**Published:** 2021-12-24

**Authors:** Lv Tong, Caiming Zhao, Zhenyin Fu, Ruiqing Dong, Zhenghong Wu, Zefeng Wang, Nan Zhang, Xinlu Wang, Boyang Cao, Yutong Sun, Dingchang Zheng, Ling Xia, Dongdong Deng

**Affiliations:** ^1^School of Biomedical Engineering, Dalian University of Technology, Dalian, China; ^2^Department of Cardiology, The First Affiliated Hospital of Soochow University, Suzhou, China; ^3^College of Biomedical Engineering and Instrument Science, Zhejiang University, Hangzhou, China; ^4^Department of Cardiology, Dushu Lake Hospital Affiliated to Soochow University, Suzhou, China; ^5^Department of Cardiology, Beijing Anzhen Hospital Affiliated to Capital Medical University, Beijing, China; ^6^Department of Radiology, Beijing Anzhen Hospital Affiliated to Capital Medical University, Beijing, China; ^7^Research Centre for Intelligent Healthcare, Faculty of Health and Life Science, Coventry University, Coventry, United Kingdom

**Keywords:** reentry, arrhythmias, computational modeling, simulation time, Gaussian mixture model method

## Abstract

Personalized cardiac modeling is widely used for studying the mechanisms of cardiac arrythmias. Due to the high demanding of computational resource of modeling, the arrhythmias induced in the models are usually simulated for just a few seconds. In clinic, it is common that arrhythmias last for more than several minutes and the morphologies of reentries are not always stable, so it is not clear that whether the simulation of arrythmias for just a few seconds is long enough to match the arrhythmias detected in patients. This study aimed to observe how long simulation of the induced arrhythmias in the personalized cardiac models is sufficient to match the arrhythmias detected in patients. A total of 5 contrast enhanced MRI datasets of patient hearts with myocardial infarction were used in this study. Then, a classification method based on Gaussian mixture model was used to detect the infarct tissue. For each reentry, 3 s and 10 s were simulated. The characteristics of each reentry simulated for different duration were studied. Reentries were induced in all 5 ventricular models and sustained reentries were induced at 39 stimulation sites in the model. By analyzing the simulation results, we found that 41% of the sustained reentries in the 3 s simulation group terminated in the longer simulation groups (10 s). The second finding in our simulation was that only 23.1% of the sustained reentries in the 3 s simulation did not change location and morphology in the extended 10 s simulation. The third finding was that 35.9% reentries were stable in the 3 s simulation and should be extended for the simulation time. The fourth finding was that the simulation results in 10 s simulation matched better with the clinical measurements than the 3 s simulation. It was shown that 10 s simulation was sufficient to make simulation results stable. The findings of this study not only improve the simulation accuracy, but also reduce the unnecessary simulation time to achieve the optimal use of computer resources to improve the simulation efficiency and shorten the simulation time to meet the time node requirements of clinical operation on patients.

## Introduction

Ventricular tachycardia (VT) is a life-threatening heart disease that occurs frequently in patients with myocardial infarction (MI), and one of the most prominent causes of sudden cardiac death (SCD) (Kusumoto et al., 2018). After acute MI, the myocardium in the center of infarct area is replaced by electric insulated fibrotic tissue, while some active myocardial cells extend into the dense fibrosis to form a slow concoction area which usually is called as gray zone (GZ). Thus, the heart tissue in patients with MI can be divided into three categories: non-infarct tissue, core scar, and GZ (Wu, 2017). The heterogeneity in the GZ slows down the electrical conduction in this part of the tissue, which in turn causes unidirectional conduction block and predisposes to reentry, leading to arrhythmias and increasing the risk of infarction in patients.

Clinical and experimental studies have shown that reentries in patients with VT can be sustained by anatomic reentry or functional reentry (Aguilar and Nattel, 2015; Martin et al., 2018). The anatomical reentry is very stable and always rotates around a fixed area (Fernández-Armenta et al., 2013; Josephson et al., 2014; Soto-Iglesias et al., 2020). For the functional reentry, the electric impulse proceeds as a single wavefront through a constrained region known as the central common pathway or isthmus of the reentrant circuit (Ciaccio et al., 2016; Martin et al., 2018; Crinion et al., 2020). In clinic, the critical isthmuses which sustain that the anatomical reentry can be measured by the high-density electroanatomic multipolar mapping system, but it is not easy to directly measure the critical isthmuses which sustain functional reentry (Martin et al., 2018; Crinion et al., 2020), especially for those unstable or non-sustained reentries (Nishimura et al., 2020).

Computational modeling has been widely used for the non-invasive investigation of lethal heart rhythm disorders and their treatment, including not only risk stratification of patients with MI (Behradfar et al., 2014; Deng et al., 2016; Lopez-Perez et al., 2019), the prediction of reentry location (Deng et al., 2015), but also guiding for VT ablation in clinic (Prakosa et al., 2018). Due to the high demand of computational resource of modeling, the reentries (both anatomic and functional) induced in the models are usually simulated for just a few seconds which is usually less than 5 s (Deng et al., 2015, 2016, 2019a,b; Arevalo et al., 2016; Prakosa et al., 2018; Ukwatta et al., 2018). In our previous work, we found that some reentries induced in the model were not stable, and become non-sustained before the end of simulation. In clinic, it is common that reentries last for more than several minutes and the morphologies of reentries are not always stable (Katritsis et al., 2012; Martin et al., 2018; Nishimura et al., 2020), so it is not clear that whether the simulation of reentries for just a few seconds is long enough to match the arrhythmias measured in clinic. Furthermore, as far as we know, still no work has been done to study whether the personalized cardiac modeling can reproduce most of the VT categories which include sustained stable reentry, sustained non-stable reentry, and non-sustained reentry measured in clinic.

In this study, we used personalized virtual heart models to: (1) find out an optimal dynamic stimulation protocol to save computational resources and make the simulation results more robust; and (2) study whether the personalized cardiac modeling can reproduce most of the VT categories measured in clinic.

## Materials and Methods

### Clinical Data

For this retrospective study, we used data from five patients who suffered from ischemic cardiomyopathy between 2018 and 2019 at Beijing Anzhen Hospital, and this study was approved by the Institutional Review Board of Beijing Anzhen Hospital. Cardiovascular magnetic resonance-late gadolinium enhancement (CMR-LGE) images of the five patients were collected and used to build the heart models. Cardiac MRI were acquired by 3.0 T scanner (Sonata, Siemens, Erlangen, Germany) or GE scanner (DISCOVERY MR 750w; GE, Boston, MA, United States). The detailed image acquisition protocol can be found in the previous published literature (Woodard et al., 2007; Klinke et al., 2013; Kramer et al., 2020). The scanning layer thickness was 8–10 mm with image resolution between 1.36 and 1.64 mm (detailed information is listed in Table 1).

**TABLE 1:** Detailed cardiovascular magnetic resonance-late gadolinium enhancement (CMR-LGE) images information of each patient.

	Size	In-plane resolution (mm)	Slice sickness (mm)	Layer count
PAT01	256 × 208	1.52344 × 1.52344	9	14
PAT02	208 × 256	1.5625 × 1.5625	8	13
PAT03	256 × 192	1.36719 × 1.36719	8.4	10
PAT04	240 × 256	1.52344 × 1.52344	10	10
PAT05	208 × 256	1.48438 × 1.48438	8	11

### Image Processing Pipeline

All analyses and measurements were manipulated on custom software developed in MATLAB (MathWorks Inc., MA, United States). The epicardial and endocardial boundaries, for each two-dimensional slice in the LGE images, were manually segmented by two experienced specialists and excluded papillary muscles from the endocardium. The part of pixels between the boundaries were considered as the myocardium. Then, the modified Gaussian mixture model (MGMM) method was used for the subsequent processing of the segmented myocardium, summarized as follows (Hennemuth et al., 2013; Pop et al., 2013).

#### Modified Gaussian Mixture Model Method

**FIGURE 1:**
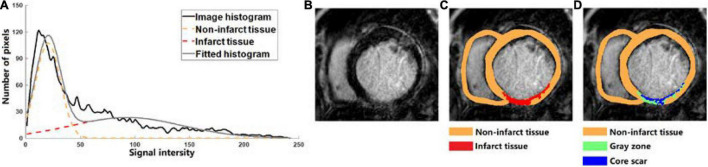
**(A)** Tissue classification example of cardiovascular magnetic resonance-late gadolinium enhancement (LGE-MRI) scanned from one human heart into two categories which are non-infarct tissue (orange dotted line) and infarct tissue (red dotted line) by using the Gaussian mixture model (GMM) method. **(B–D)** Based on the LGE-MRI of patient, the myocardial tissue was divided into non-infarct region (orange) and infarcted region (red) by GMM method, and the infarcted region was further divided into gray zone (GZ) (green) and core scar (blue).

A classification method based on Gaussian mixture model (GMM) was used to segment the tissue inside the epi- and endocardium boundaries. GMM assumes that the image intensity of each class of tissues obeys a Gaussian distribution, where each class has its own mean intensity and variance, and classifies the tissues into different classes by best fitting the image histogram based on the expectation maximization method (Figure 1A). One slice of the original CMR-LGE images was shown in Figure 1B. Then, two different categories that included non-infarct and infarct tissue were obtained (Figure 1C). Next, the maximal component in each layer and the components with pixels more than 50% of the maximal components were kept. Finally, the regions in each image layer with more than 15 pixels were retained to remove small clusters of pixels affected by noise or blood vessels. The detailed process of the MGMM method can be found in our previously published paper (Wu et al., 2021).

To further segment the infarct tissue detected by the MGMM-based classification method and Threshold method into GZ and core scar, the maximal (intensity_*max*_) and minimal (intensity_*min*_) values of the pixels in the infarct tissue were calculated, then the pixels > (intensity_*max*_ – intensity_*min*_) × 50% were assigned as core scar, and the rest pixels in the infarct area were assigned as GZ (Figure 1D).

### Model Construction and Simulation Protocol

**FIGURE 2:**
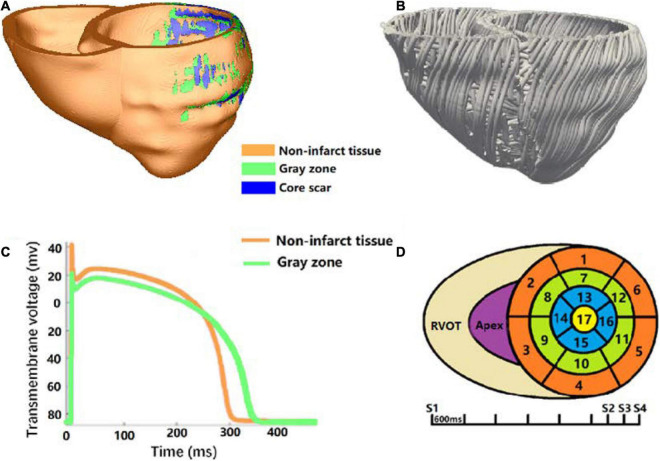
Virtual-heart arrhythmia risk predictor methodology. **(A)** High-resolution ventricular structure model segmented into normal tissue, GZ, and core scar; **(B)** visualization of myocardial fiber orientations; **(C)** action potential for non-infarcted tissue (orange) and GZ (green); **(D)** virtual-heart arrhythmia risk predictor pacing sites.

After image segmentation, CardioViz3D (Toussaint et al., 2008) (INRIA, Sophia Antipolis, France) was used to interpolate the segmented low-resolution images to high-resolution images (about 0.4 mm). The 3D geometry of the infarct tissue which includes core scar and GZ was reconstructed using log odds method (Ukwatta et al., 2015), and merged with the corresponding ventricular high-resolution images. For each patient-specific bi-ventricular geometry, the commercial software Mimics Innovation Suite (Materialise NV, Leuven, Belgium) was used to generate the finite-element mesh (Figure 2A). The target average edge lengths were about 400 mm. Fiber orientations in the mesh were assigned using a previously validated rule-based method (Bayer et al., 2012). It uses the Laplace–Dirichlet method to define transmural and apicobasal directions at every point in the ventricles, and then employs the bi-directional spherical linear interpolation to assign fiber orientations based on experimental measured angles (Eggen et al., 2012; Lombaert et al., 2012) (−40 to +65° from epi- to endocardium) (Figure 2B).

Electrophysiology properties were assigned in the model as previously described (Prakosa et al., 2018; Deng et al., 2019a). Briefly, non-infarcted tissue was assigned with the human ventricular myocyte action potential cell model of ten Tusscher et al. (2004; Figure 2C). Action potential remodeling in the GZ based on experimental recordings was implemented as follows (Arevalo et al., 2013; Deng et al., 2016): peak sodium current, peak L-type calcium current, peak potassium currents IKr, and IKs were decreased to 38, 31, 30, and 20% of the original values in the Ten Tusscher model, respectively. Core scar was modeled as passive tissue. Conductivity values were assigned such that resultant conduction velocities (0.60 m/s in non-infarct tissue and 0.30 m/s in the GZ) were approximate to that measured clinically in the model, as illustrated in Anter et al. (2016).

The propagation of electrical activity in the heart model was simulated by solving a reaction-diffusion partial differential equation with finite-element method (Plank et al., 2008). Simulations of electrical activity in the patient-specific heart models with Neumann boundary conditions were executed in a monodomain representation of the myocardium using the openCARP simulation environment (Plank et al., 2021)^1^ on high performance computers at Dalian University of Technology, China. Programmed electrical stimulation used in the previously published articles (Prakosa et al., 2018; Deng et al., 2019a) was used to induce VTs in the models of five patients. In brief, the protocol was consisted with 6 beats of 600 mm cycle length (S1), followed by premature stimulus (S2) at 90% of the S1 cycle length. The time between S1 and S2 was gradually shortened until VT was induced. If VT was not induced, a second premature stimulus was performed after S2. If VT was still not induced, a third premature stimulus was performed after S3. Furthermore, all models were paced from 19 ventricular sites, such as 17 sites on the LV (left ventricular), 1 near the right ventricular outflow tract, and 1 at the right ventricle apex, according to American Heart Association Classification Standards (Cerqueira et al., 2002; Figure 2D). After reentry was induced, 10 s of VT were simulated to detect the presence of arrhythmia. The VT morphology and location at the end of 3 and 10 s were analyzed and compared.

## Results

Table 2 summarized the volumes of normal myocardium, GZ, and core scar in the reconstructed heart models from the MRI images of 5 patients. The mean ventricular volume was 176.4 cm^3^. The percentage of volume in the non-infarcted ventricular myocardium had a range from 86.4%∼96.4%, while the percentage of GZ volume and core scar ranged from 2.8%∼7.6% and 0.8%∼7.1%, respectively.

**TABLE 2:** Summary volume database of five patients.

Model	Normal tissue (cm^3^)	% of total volume	GZ (cm^3^)	% of total volume	Core scar (cm^3^)	% of total volume	Total volume of heart
PAT01	208.1	94.8	6.7	3.0	4.9	2.2	219.7
PAT02	175.2	86.4	13.2	6.5	14.3	7.1	202.7
PAT03	164.1	93.8	6.6	3.8	4.3	2.4	175.0
PAT04	230.6	96.4	6.6	2.8	1.9	0.8	239.1
PAT05	104.2	87.3	9.0	7.6	6.1	5.1	119.3
Mean ± SD	176.4 ± 48.2	91.7 ± 4.6	8.4 ± 2.9	4.7 ± 2.2	6.3 ± 4.7	3.5 ± 2.5	191.2 ± 46.6

Our simulation results showed that 59.0% (23/39) of VTs induced in the 3 s simulation continued propagating to the end of 10 s simulation, but some of the induced VT morphologies had great difference before and after 3 s simulation. We divide the VTs into four different types based on the stable time of the final VT morphology (Tables 3, 4).

**TABLE 3:** Ventricular tachycardias (VTs) induced in all 5 models with simulation time of 3 and 10 s.

	3 s simulation	Reentry morphology	10 s simulation	Reentry morphology	Total VTs	Percentage
Type 1	Sustained reentry	Not stable reentry	Sustained reentry	Stable reentry	10	25.6%
Type 2	Sustained reentry	Not stable reentry	Sustained reentry	New stable reentry	4	10.3%
Type 3	Sustained reentry	Stable reentry	Sustained reentry	Same stable reentry	9	23.1%
Type 4	Sustained reentry	Stable reentry	Non-sustained reentry	No reentry	16	41.0%

**TABLE 4:** Simulation time statistics when 39 stimulus sites induced reentry reached stability.

Patient number	Type 1		Type 2		Type 3		Type 4	
	Number of VTs	Time of VTs to be stable	Number of VTs	Time of VTs to be stable	Number of VTs	Time of VTs to be stable	Number of VTs	Time of VTs to be disappeared
PAT01	1	2.35 s	\	\	\	\	1	3.9 s
PAT02	3	2.6–2.9 s	3	4.1–7.3 s	5	1.46–2.3 s	\	\
PAT03	1	2.5 s	1	3.3 s	4	1.4–2.3 s	6	3.09–8.9 s
PAT04	\	\	\	\	\	\	4	3.06–5.3 s
PAT05	\	\	\	\	\	\	5	3.02–8.31 s
Total	10	2.35–2.9 s	4	3.3–7.3 s	9	1.4–2.3 s	16	3.02–8.9 s

*“\” indicates that no corresponding type of VT was induced in the model, and thus the “Time of VTs to be stable” was not applicable.*

For type 1 VT, the VT location and morphology was not stable, it changed location in 1–3 cycles, but in the last 2–3 cycles, the VT location and morphology became stable. And in the extended 10 s simulation, the VT location and morphology was the same as the VT at the end of 3 s simulation. About 25.6% (10/39) VTs were type 1 reentry, and the time of VTs to be stable varied from 2.35 to 2.9 s.

**FIGURE 3:**
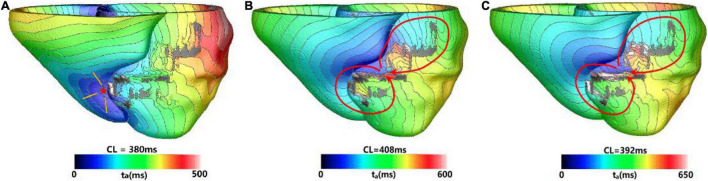
Example of a reentry morphology for type 1 ventricular tachycardia (VT). **(A)** Showed breakthrough on the heart surface of a reentry induced in a model before stable. **(B)** Showed the reentry morphology at the end of 3 s. **(C)** Reentry morphology induced in the same model at the end of 10 s. Red arrowhead indicates the direction of VT propagation. Red star indicates the VT breakthrough on epicardium and the arrowheads indicate the direction of electrical propagation. The color scales in **(A,B)** indicate activation times and the black areas represent core scar—there is no electrical propagation there.

Figure 3 showed one example of type 1 VT. The initial reentry took place in the front of mid anterior cavity of endocardium isthmus, and the breakthrough (Figure 3A) presented here was transmitted from epicardium, where the reentrant loop was large and lasted for 5 cycles. While the simulation reached the end of 3 s, a stable “figure of eight” reentry propagated two cycles at the middle part of anterior wall (Figure 3B). As shown in Figure 3C, with the extended 10 s simulation, the reentry kept rotating without any changes.

For type 2 VT, the VT location and morphology was not stable, it changed locations during the entire 3 s simulation. But in the extended 10 s simulation, the VT location stabilized to a fixed location and the morphology did not change. About 10.3% (4/39) VTs were type 2 reentry, and the time of VTs to be stable varied from 3.3 to 7.3 s.

**FIGURE 4:**
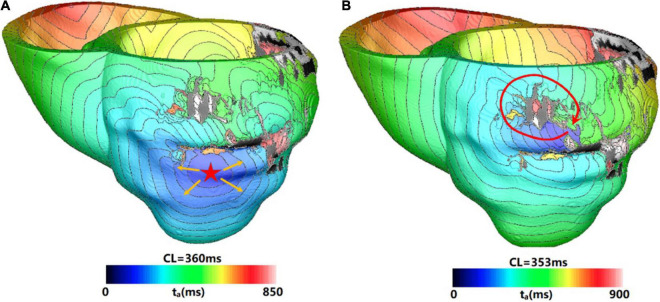
Example of a reentry morphology for type 2 VT. **(A)** Reentry morphology induced in a model at the end of 3 s. **(B)** Reentry morphology induced in the same model at the end of 10 s. Red star indicates the VT breakthrough on epicardium and the arrowheads indicate the direction of electrical propagation. The color scales in **(A,B)** indicate activation times and the black areas represent core scar—there is no electrical activation there.

Figure 4 showed one example of type 2 VT. During the 3 s simulation, several reentries were observed between myocardium and epicardium. The electrical propagation progress began with 3 cycles of one reentry with breakthrough at the epicardial surface. Then, the reentry changed to the anterior basal part of LV, and followed by changing to a new location with 2 cycles (Figure 4A). In the extended 10 s simulation, the chaotic reentry disappeared, and a new stable reentry formed at the anterior wall which lasted to the end of 10 s simulation (Figure 4B).

For type 3 VT, the VT location and morphology was stable in the 3 s simulation, and it did not change location and morphology in the extended 10 s simulation. Approximately, 23.1% (9/39) VTs were type 3 reentry, and the time of VTs to be stable varied from 1.4 to 2.3 s.

**FIGURE 5:**
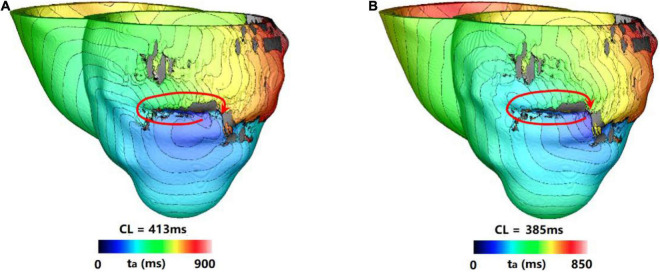
Example of a reentry morphology for type 3 VT. **(A)** Reentry morphology induced in a model at the end of 3 s. **(B)** Reentry morphology induced in the same model at the end of 10 s. Red arrowhead indicates the direction of VT propagation.

Figure 5 showed one example of type 3 VT. Figure 5A showed one reentry induced in a model in the 3 s simulation. The reentry located at the middle part of anterior wall, and it was a circulator. When the simulation was extended, there were little changes of morphology and position over time.

For type 4 VT, the VT location and morphology may change or be stable at the end of 3 s simulation, but they disappeared in the extended 10 s simulation. About 41.0% (9/39) VTs were type 4 reentry, and the time of VTs to be disappeared varied from 3.02 to 8.9 s.

**FIGURE 6:**
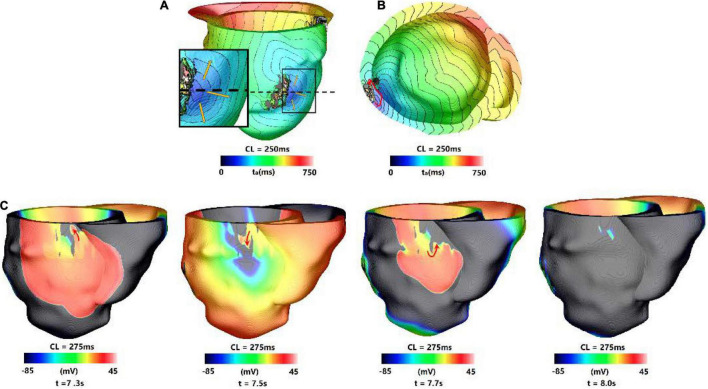
Example of a reentry morphology for type 4 VT. **(A)** Reentry morphology induced in a model at the end of 3 s, yellow arrow indicates the direction of propagation. **(B)** The entire reentry conduction pathway as shown in the **(A)**. **(C)** Electrical propagation of the reentry in 10 s simulation. Red arrows show direction of propagation. The color bar shows the transmembrane potentials, and the instant time (t) of each map is shown below.

Figure 6 showed one example of type 4 VT. The reentry induced in the 3 s simulation disappeared in the extending 10 s simulation. In the 3 s simulation, the reentry propagated from epicardial surface into the interior wall and backed to the surface. A total of six reentry cycles occurred, as shown in Figures 6A,B. When the simulation time was extended, the reentry at middle part of LV lateral wall in the 3 s simulation disappeared. A new reentry emerged at the posterior basal wall, and it lasted for six cycles and then disappeared (Figure 6C).

Table 5 summarized the clinical measurements and the 3 and 5 s simulation results of all five patients. For patient 1, the clinical diagnosis showed that there was no VT event when the patient was in hospital. Two sustained VTs were induced in the corresponding model of patient 1 in the 3 s simulation. In the 10 s simulation, 1 VT was terminated and the other survived to 10 s. Although 1 VT was induced in the 10 s simulation, the VT inducing ratio (5.3%) was very low, suggesting that the patient had a low probability to induce VT. Therefore, the result of 10 s simulation matched better than the 3 s simulation with the clinical diagnosis.

**TABLE 5:** Comparison of 3 and 10 s simulation results of modified Gaussian mixture model (MGMM) model with clinical data.

	Clinical measurements		Simulation results of MGMM method					
	VT in clinic	VT location	Results in 3 s simulation			Results in 10 s simulation		
			Number of VTs	Number of VTs related to clinic	Induction ratio	Number of VTs	Number of VTs related to clinic	Induction ratio
PAT01	N	–	2	–	10.5% (2/19)	1	–	5.3% (1/19)
PAT02	Y	Middle lateral wall of LV	3	1	57.9% (11/19)	2	1	57.9% (11/19)
PAT03	Y	Close to apex	3	1	63.2% (12/19)	1	1	31.6% (6/19)
PAT04	N	–	1	–	21.1% (4/19)	0	–	0% (0/19)
PAT05	N	–	2	–	26.3% (5/19)	0	–	0% (0/19)

*Number of VTs indicates the number of sustained VT morphology induced in the model. Induction ratio refers to the proportion of pacing sites induced sustained reentry in the 19 pacing sites.*

For patient 2, VT events were monitored four times and the location of VT was at the middle lateral wall of the LV. There were three different VTs induced in the 3 s simulation, one VT was related to the clinical measured location. The rest 2 VTs were not related to the clinical location. Two different VTs were induced in the 10 s simulation, one VT induced by 10 of 19 pacing sites was related to the clinical measured location. The rest one induced in only 1 pacing site was in another location.

For patient 3, VT events were monitored and the VT was epicardial reentry close to apex. Three sustained VTs were induced in the corresponding model of patient 3 in the 3 s simulation. One was related to the clinical measurement, and the other two VTs induced in 6 pacing sites were not related to the clinical location and terminated in the 10 s simulation. There was only one sustained VT induced in 6 pacing sites in the 10 s simulation which was related to the clinical measurement.

For patient 4, the clinical diagnosis showed that there was no infarct related VT event when the patient was in hospital, and this patient only had ventricular premature beat. There was one sustained VT induced in 4 pacing sites in the 3 s simulation, but the VT only lasted to maximum 5 s in the 10 s simulation. There was no sustained reentry induced in the 10 s simulation, it was corresponded to the clinical diagnosis.

For patient 5, the clinical diagnosis showed that there was non-sustained VT event when the patient was in hospital, and VT was not inducible in this patient during implantable cardioverter defibrillator (ICD) implantation procedure. There were 2 sustained VTs induced in 5 pacing sites in the 3 s simulation, but all these VTs terminated in the 10 s simulation. One VT lasted to 8.3 s in the 10 s simulation, this was corresponding to the clinical diagnosis of non-sustained VT.

Based on the comparison of results in 3 and 10 s simulation with clinical measurements (as shown in Table 5), the results showed that sustained VTs induced in 16 pacing sites in 3 s simulation were terminated in the 10 s simulation, this meant that 41% (16/39) of the sustained VTs in the 3 s simulation became non-stained in the 10 s simulation. The clinical measurements of patient 4 and 5 showed that the simulation results in 10 s simulation were more accurate than the 3 s simulation. In the 3 s simulation, more VTs were induced than the 10 s simulation, and some of VTs were not corresponding to clinical measurements.

## Discussion

In this study, we used personalized computational MI models which were reconstructed from LGE-MRI images to observe how long simulation of the induced arrhythmias in the personalized cardiac models is sufficient to get a stable location and morphology, and whether these characteristics have difference with varied simulation time. Based on the simulation results, we found that 41% of the sustained reentries in the 3 s simulation group terminated in the longer simulation groups (10 s). The second finding in our simulation was that only 23.1% of the sustained reentries in the 3 s simulation did not change location and morphology in the extended 10 s simulation. The third finding is that 35.9% reentries were not stable when using 3 s simulation, which means that an extended simulation time is necessary.

For type 1 and 2 VT, because it is not stable when the simulation time is over, we should extend the simulation to a longer time to make sure the reentry become stable. But it is hard to give a consistent value for the simulation time based on the large variation of VT termination time. It should be possible to use a dynamic simulation time for each induced reentry, that is, 3, or 5, or 7, until 10 s. This will not only save the simulation resources, and make the simulation results more robust. These two types of VT may be related to the clinical hemodynamically unstable tachycardia (Nishimura et al., 2020). These kind of VTs are more likely sustained by functional reentry, this means that the involved VT is sustained by reentries through conduction channels around an area of functional block (Aguilar and Nattel, 2015; Deng et al., 2019a). The morphology and location of this kind of reentry were complex. The unstable characteristics of functional reentry was due to the intrinsic nature of the cardiac single cells (action potential duration adaptation, conductivity adaptation, etc.) (Chang et al., 2009; Lawson et al., 2020).

For type 3 VT, because the reentry gets stable at very early stage, and it does not change location and morphology in the extended simulation, thus for these reentries 3 s simulation is sufficient to get a robust result. This will save a lot of simulation resources and analysis time. This kind of VT is more likely sustained by anatomical reentry (Aguilar and Nattel, 2015), this means that the VT is sustained by reentries through conduction channels surrounded by scar on all sides. This kind of reentry was reported in other personalized heart modeling (Deng et al., 2019a; Kruithof et al., 2021) and clinical measurements (Anter et al., 2016; Nishimura et al., 2020). Our simulation results showed that type 3 VT was very stable, its morphology and location did not change during the entire 10 s simulation after reentry was initiated.

For type 4 VT, because the reentry gets disappeared in the extended simulation, this will not only spend more simulation resources to get robust results, but also make the results analysis more complex. It is very hard to recognize this kind of reentry as sustained reentry or non-sustained reentry as compared with the clinal definition (Katritsis et al., 2012). Besides, here rises a question that should this reentry be treated as an ablation target? These questions will be studied in the future when the clinical data of these patients become available.

For type 1, 2, and 4 VTs, the simulation results showed that simulation time must be extended to get the final stable reentry. Furthermore, the sustained reentry in 3 s simulation may become non-sustained reentry (or disappear in other word), this is consistent with clinical measurements that some reentries were unstable and may disappear in a few seconds (Ukwatta et al., 2015). In addition, the simulation results showed similar findings that no big morphological differences in circuit dimensions between stable and unstable VTs with the clinical measurements (Nishimura et al., 2020). We found that the CL of non-sustained reentry (type 4 VT) is shorter than other type of reentry, and the CL of type 1, 2, and 4 VTs did not have significant differences.

## Conclusion

In the current work, we not only quantitatively analyzed the commonly clinical measured phenomena which were some arrhythmias lasted for more than several minutes but others lasted only a couple of cycles or seconds, and some arrhythmias had no stable morphology or location, and the morphologies of reentries were not always stable, but also proposed a dynamic stimulation protocol to save computational resources and make simulation results more robust. We believe that the findings of this study not only improve the simulation accuracy, but also reduce unnecessary simulation time to achieve the optimal use of computer resources, thus improve the simulation efficiency and shorten the simulation time to meet the time node requirements of clinical operation on patients.

## Limitations

One limitation of the current study is the small sample size of five patients, but it could demonstrate the findings in the conclusion section. Another limitation is that we did not compare the non-sustained reentry with the clinical definition, but we have compared the simulation results with clinical diagnosis. We define the sustained and non-sustained reentry only based on the lasting time of reentry within 10 s. We will improve the comparison when we get available clinical data in the future. The third limitation is that we have not done the quantitative comparison of VT characteristics in the model with clinical data, this is because that we do not have the corresponding patient data. But our simulation results can reproduce a lot of clinical measured phenomenon, this demonstrate that the personalized virtual heart simulation approach may provide a useful tool to help in understanding the underling mechanism of VT and assist in clinical decisions to identify and ablate the reentrant circuit(s).

## Data Availability Statement

The original contributions presented in the study are included in the article/supplementary material, further inquiries can be directed to the corresponding author/s.

## Ethics Statement

The studies involving human participants were reviewed and approved by the Beijing Anzhen Hospital. Written informed consent for participation was not required for this study in accordance with the National Legislation and the Institutional Requirements.

## Author Contributions

CZ, RD, ZFW, NZ, and XW provided the human MRI and clinical data. DD, CZ, and LX designed the idea of the manuscript. ZF, ZHW, LT, NZ, BC, and YS did the manual segmentation. ZF, LT, ZHW, BC, and YS generated the figures. ZF, LT, DD, DZ, and LX wrote and revised the manuscript. All authors discussed the results and commented on the manuscript.

## Conflict of Interest

The authors declare that the research was conducted in the absence of any commercial or financial relationships that could be construed as a potential conflict of interest.

## Publisher’s Note

All claims expressed in this article are solely those of the authors and do not necessarily represent those of their affiliated organizations, or those of the publisher, the editors and the reviewers. Any product that may be evaluated in this article, or claim that may be made by its manufacturer, is not guaranteed or endorsed by the publisher.

## Footnotes

1
https://opencarp.org/
